# Tetrandrine Derivatives as Promising Antibacterial Agents

**DOI:** 10.1021/acsomega.3c01368

**Published:** 2023-07-26

**Authors:** Viviana I. Calvillo-Páez, Maribel Plascencia-Jatomea, Adrián Ochoa-Terán, Carmen L. Del-Toro-Sánchez, Ricardo I. González-Vega, Sandra M. González-Martínez, Karen Ochoa Lara

**Affiliations:** † Centro de Graduados e Investigación en Química, Tecnológico Nacional de México, Campus Tijuana, CP 22444 Tijuana, B.C., México; ‡ Departamento de Investigación y Posgrado en Alimentos, Universidad de Sonora, Rosales y Encinas s/n, Col. Centro, CP 83000 Hermosillo, Sonora, México; § Departamento de Investigación en Polímeros y Materiales, Universidad de Sonora, Rosales y Encinas s/n, Col. Centro, CP 83000, Hermosillo, Sonora, México

## Abstract

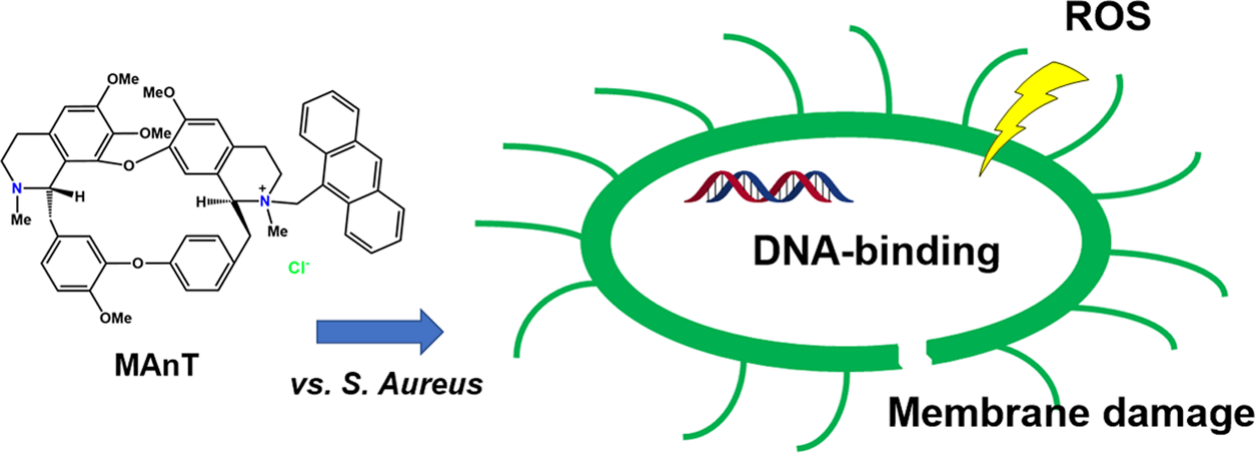

This work reports on the antibacterial activity of two tetrandrine derivatives, with acridine (**MAcT**) and anthracene (**MAnT**) units, against Gram-positive and Gram-negative bacteria of clinical importance by the broth microdilution method as well as their antioxidant activity against ABTS^•+^ and DPPH^•+^ radicals. Unlike natural tetrandrine, its derivatives inhibited bacterial growth, showing selectivity against *Staphylococcus aureus* with notable activity of **MAnT** (MIC = 0.035 μg/mL); this compound also has good activity against the ABTS^•+^ radical (IC_50_ = 4.59 μg/mL). Cell membrane integrity studies and reactive oxygen species (ROS) detection by fluorescent stains helped to understand possible mechanisms related to antibacterial activity, while electrophoretic mobility assays showed that the derivatives can bind to bacterial DNA plasmid. The results indicate that **MAnT** can induce a general state of oxidative stress in *S. aureus* and *Escherichia coli*, while **MAcT** induces an oxidative response in *S. aureus*. Complementary electrochemical studies were included.

## Introduction

The indiscriminate use of antibiotics has led to the emergence of multidrug-resistant strains in some bacterial species, which has become a major threat to human health.^1^ The group of frequently multidrug-resistant bacteria, termed ESKAPE pathogens by the Infectious Diseases Society of America, are *Enterococcus faecium*, *Staphylococcus aureus*, *Klebsiella pneumoniae*, *Acinetobacter baumannii*, *Pseudomonas aeruginosa*, and Enterobacter species.^2^ Methicillin-resistant *S. aureus* (MRSA) and multidrug-resistant Gram-negative bacteria, such as *Escherichia coli*, are among the most prevalent, causing a number of serious and difficult-to-treat nosocomial infections.^3^ For this reason, new strategies are necessary both for the management of antibiotics and for the development of new antibacterial agents with fewer side effects.^4^ Among the natural sources, animal and vegetal extracts, metabolites of microbial origin, e.g., defense enzymes, and organic acids, and secondary metabolites from plants, such as phytoalexins, saponins, terpenoids, phenolics, alkaloids, etc., have been successfully tested as potential antibacterial agents.^5−7^ In this regard, alkaloids are a group of promising antibacterial agents, as they can be recovered from many sources and have a broad antibacterial spectrum with good activity against common clinical strains, including multidrug-resistant bacteria. Within this group, the isoquinoline alkaloids, those with an isoquinoline skeleton, have been very attractive to the scientific community and have therefore been extensively studied.^6^ The *S*,*S*-(+)-tetrandrine is a bisbenzylisoquinoline alkaloid that has diverse biological properties, being the antiproliferative activity in various cancer cell lines one of the most relevant, and has been extensively studied in the past decade.^6,8−12^ In addition, this alkaloid has been studied for the treatment of COVID-19, demonstrating in various clinical studies to be effective in improving the prognosis of patients and to have the potential to reduce the entry of SARS-CoV-2S pseudovirions.^13,14^

Also, the antibacterial and antioxidant activity of tetrandrine has been reported, although these two properties have been poorly explored.^15−26^ Regarding its antioxidant activity, tetrandrine is capable of trapping superoxide anion radicals at a concentration of 0.1 μg/mL, while at a concentration of 10 μg/mL, it causes a decrease in the consumption of hexose-monophosphate and hydrogen peroxide production. The link between the antioxidant capacity of this alkaloid and some of its pharmacological effects has been previously evidenced.^16−19^ Regarding the antibacterial activity of tetrandrine, it has been shown to have a synergistic effect with some antibiotics such as isoniazid and ethambutol, commonly used for the treatment of *Mycobacterium tuberculosis*.^20^ Likewise, there are reports showing that tetrandrine has an inhibitory effect against methicillin-resistant *S. aureus* and *E. coli* at a concentration of 125–250 μg/mL, observing a synergistic effect on these strains when using mixtures of tetrandrine with various antibiotics.^4,6,15,21^ More recently, the inhibitory effect of this alkaloid against Gram-negative bacteria such as MβL *P. aeruginosa* and *K. pneumoniae* and the synergistic effect between colistin and tetrandrine against resistant *Salmonella* have been demonstrated.^21,23^ In addition, the antifungal activity of tetrandrine against *Candida albicans*, *Aspergillus fumigatus*, and other species of dermatophytes has been reported.^24−26^

**Figure 1:**
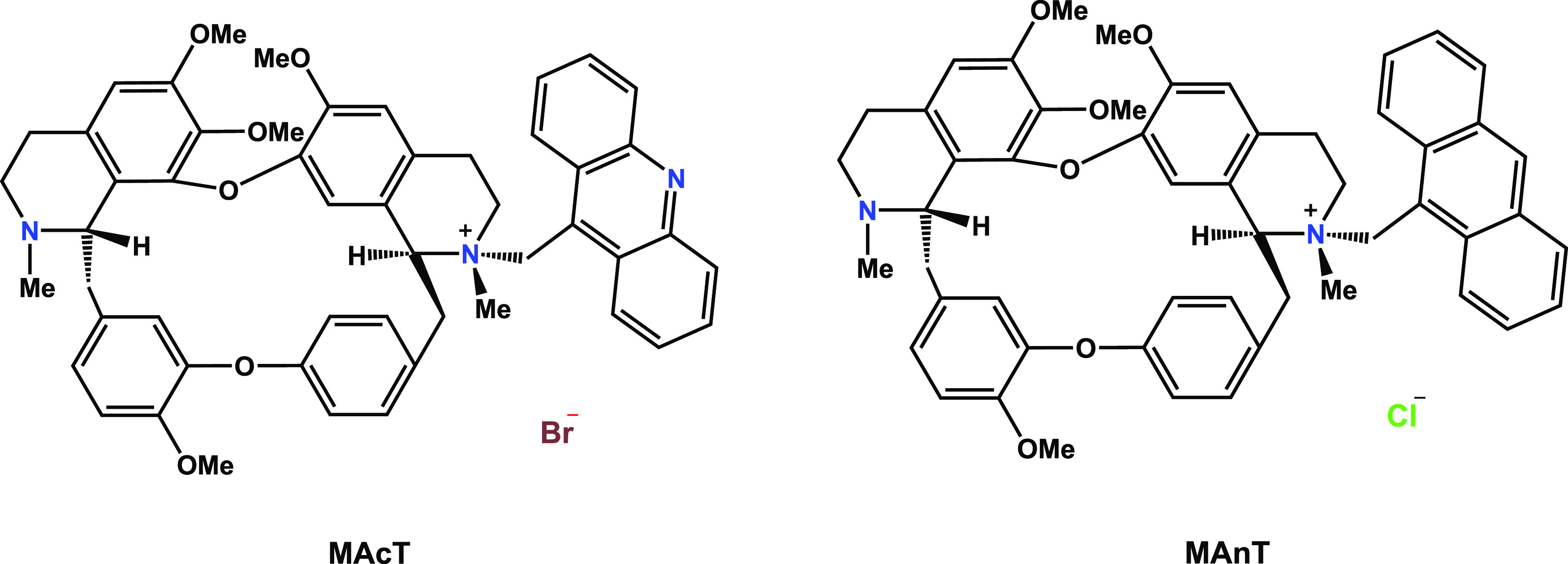
Chemical structures of mono-*N*-substituted tetrandrine derivatives **MAcT** and **MAnT** studied in this work.

The chemical modification of tetrandrine should be a good strategy to improve its biological properties. In this sense, there are some works about tetrandrine derivatives with antiproliferative activity against various tumor cell lines.^9−11^ However, to the best of our knowledge, there are no reports documenting other biological properties of tetrandrine derivatives. Our research group is a pioneer in the chemical modification of the tetrandrine alkaloid, based on the alkylation of its nitrogen atoms, as well as in the physicochemical and molecular recognition studies of this type of derivatives toward anions of biological importance, such as DNA.^27−35^ More recently, we have also studied the antiproliferative activity in various cancer cell lines for some of these derivatives.^32,35^ In this context, the mono-*N*-substituted derivatives with acridine and anthracene **MAcT** and **ManT**, respectively (Figure 1),^32,33^ were shown to have good antiproliferative activity, with an outstanding IC_50_ of 2.74 μg/mL for **ManT** against HeLa cervical cancer cell line, a value 3.3 times lower than that of natural tetrandrine. Furthermore, it was also shown that both derivatives have good affinity toward ds-DNA under physiological conditions.

Considering all of the above, here we report the antibacterial and antioxidant activity evaluation of natural tetrandrine and its derivatives **MAcT** and **ManT**. To explore the possible mechanisms related to its antibacterial activity, cell membrane integrity studies and detection of reactive oxygen species through fluorescent staining, electrophoretic mobility assays, and electrochemical studies were also included. The results presented here show that systematic modifications in natural tetrandrine improve its biological activity and that this strategy is a good option to develop new drugs and effective antibacterial agents.

## Results and Discussion

### Antibacterial Activity

The antibacterial activity of tetrandrine and its derivatives **MAnT** and **MAcT** was tested against *S. aureus* ATCC 25923, *E. coli* ATCC 25922, *K. pneumoniae* ATCC 13883, and *P. aeruginosa* ATCC 10145. The 96-well plate broth microdilution technique was used, as described in the Experimental Section; DMSO with culture medium, ampicillin (10 and 30 μg/mL) and chloramphenicol (30 μg/mL) were used as negative and positive control samples. Figure S1 (Supporting Information) shows the graphs of the CFU/mL × 10^8^ against the concentration in μg/mL, while Tables 1 and S1 summarize the results found in MIC, and IC_50_/IC_99_ values, respectively. As can be seen in both tables, the MIC and IC_50_ values of tetrandrine could not be determined for any bacterial strain since the alkaloid did not show antibacterial activity against the species studied; with growth of microorganisms in all concentrations tested. In contrast, **MAnT** antibacterial activity was found against *S. aureus*, *E. coli*, and *K. pneumoniae*, with a particularly greater inhibitory effect (*P* < 0.05) on *S. aureus*, while **MAcT** showed activity against the strains of *S. aureus* and *K. pneumoniae*, so in these cases the MIC and IC_99_ values could be obtained. It is important to mention that under the experimental conditions used in this work, it was not possible to analyze the effect of the compounds against *P. aeruginosa*. This can be attributed to the fact that *P. aeruginosa* is a bacterium capable of forming biofilms, composed mainly of exopolysaccharides, extracellular DNA, and polypeptides that together form a highly hydrated polar mixture. This biofilm allows bacteria to be resistant to various antibacterial agents and therefore to proliferate abundantly (Figure S1d).^36^

**Table 1:** Minimum Inhibitory Concentration (MIC) Values of Tetrandrine and Its Derivatives against ATCC Strains Compared to Those Reported for Standard Antibiotics^a^

MIC (μg/mL ± SD)			
	*S. aureus* ATCC 25923	*E. coli* ATCC 25922	*K. pneumoniae* ATCC 13883
MAcT	6.107 ± 0.468^a^	ND*	3.210 ± 0.537^b^
MAnT	0.035 ± 0.027^b^	23.511 ± 1.262^a^	6.784 ± 0.561^a^
Tetrandrine	ND*	ND*	ND*
Ciprofloxacin^37−39^	0.040	0.210 ± 0.087	0.063
Ampicillin^40−42^	4	4	64
Chloramphenicol^39−41^	75	4	4

a Significant differences (*p* < 0.05) are indicated by different letters (a and b), one-way ANOVA. Values are mean ± standard deviation (SD) of three repetitions (*n* = 3). DMSO with culture medium, ampicillin (10 and 30 μg/mL), and chloramphenicol (30 μg/mL) were used as control samples. ND* = MIC value was not obtained at the maximum concentration used (200 μg/mL).

As mentioned, the most outstanding activity was obtained with **MAnT** against *S. aureus* with a notable MIC of 0.035 μg/mL, being considerably lower than that reported for tetrandrine (250 μg/mL).^15^ In this sense, if we compare the MIC values of known antibiotics for the same strain of *S. aureus* such as ciprofloxacin (0.04 μg/mL), as well as ampicillin (4 μg/mL) and chloramphenicol (75 μg/mL) that were used as positive controls, evidently **MAnT** is superior in activity.^37−42^

After the MIC values were obtained by optical density, the resazurin dye was used to determine bacterial viability. The fluorescence emission or colorimetric response generated by the assay is proportional to the number of live cells in the sample. Therefore, wells that appear purple or blue are indicative of the absence or loss of viability of the bacteria, while the pink wells indicate cell viability despite the presence of bioactive compounds.^43,44^

Four representative plates of this experiment are shown in Figure S2, one for each strain. The first line of wells A1–A6 represent culture medium and solvent controls: where the first three wells have only BHI culture medium, noticing the blue/purple color of the dye, and subsequently in all cases the three pink wells correspond to the culture medium with the respective bacterial inoculum, and the remaining 6 wells (A7–A12) contain DMSO as a negative control. In rows B and C of all plates, the culture medium is presented with the inoculum and increasing concentrations of tetrandrine, where a pink color can be observed, which indicates that the bacteria are still viable to reduce the dye. However, in the culture media where the tetrandrine derivatives were added, differences in the viability of each strain were observed: the wells in rows D and E contain increasing concentrations of **MAcT**, while the wells in rows F and G contain **MAnT**. When the effect on *S. aureus* was analyzed (Figures S2a and S1a), an inhibitory effect of **MAcT** (from 50 μg/mL) and **MAnT** (from 12 μg/mL) was observed, while in the case of *E. coli*, inhibitory activity was found only for **MAnT** at concentrations higher than 100 μg/mL, observing that the cells were viable up to 25 μg/mL of **MAnT**.

**Figure 2:**
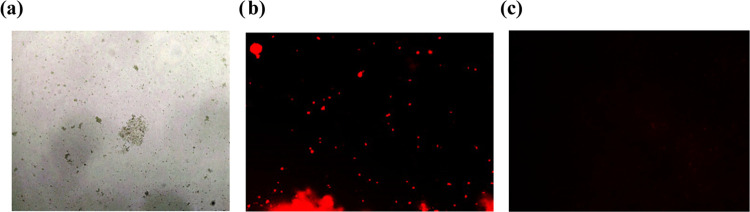
**MAnT** activity against *S. aureus* (1 × 10^8^ CFU/mL) stained with propidium iodide. Observation in an inverted microscope (40×): (a) Clear field; (b) **MAnT** (200 μg/mL); (c) negative control (*S. aureus* with culture medium).

Bacterial viability measurements are applied in various fields of research and industry, and propidium iodide (PI) is commonly used as an indicator of dead cells.^45^ PI dye emits red fluorescent when it binds to DNA due to damage to the cell membrane.^46^Figure 2 shows images of *S. aureus* cells in culture medium added with **MAnT** exposed for 24 h and stained with propidium iodide, where many red-stained cells can be observed. This observation is consistent with the viability results obtained with the resazurin dye, indicating that this tetrandrine derivative is the most active against this strain. In addition, the **MAcT** derivative exhibited a similar effect, suggesting that Gram-positive bacteria, such as *S. aureus*, have a higher susceptibility to these types of compounds. Figure S3 shows the other bacterial strains exposed to the compounds and highlights that in the case of *E. coli* the behavior is like that observed for **MAnT** against *S. aureus*.

Similar observations have been reported in the literature, related to the fact that some flavonoid and alkaloid derivatives have shown antibacterial activity against *S. aureus* and *P. aeruginosa*, which could be correlated with bacterial membrane alterations.^47^ On the other hand, natural tetrandrine did not affect the integrity of the membrane of the bacteria studied, further confirming the results of microbial growth measured through optical density values.

**Figure 3:**
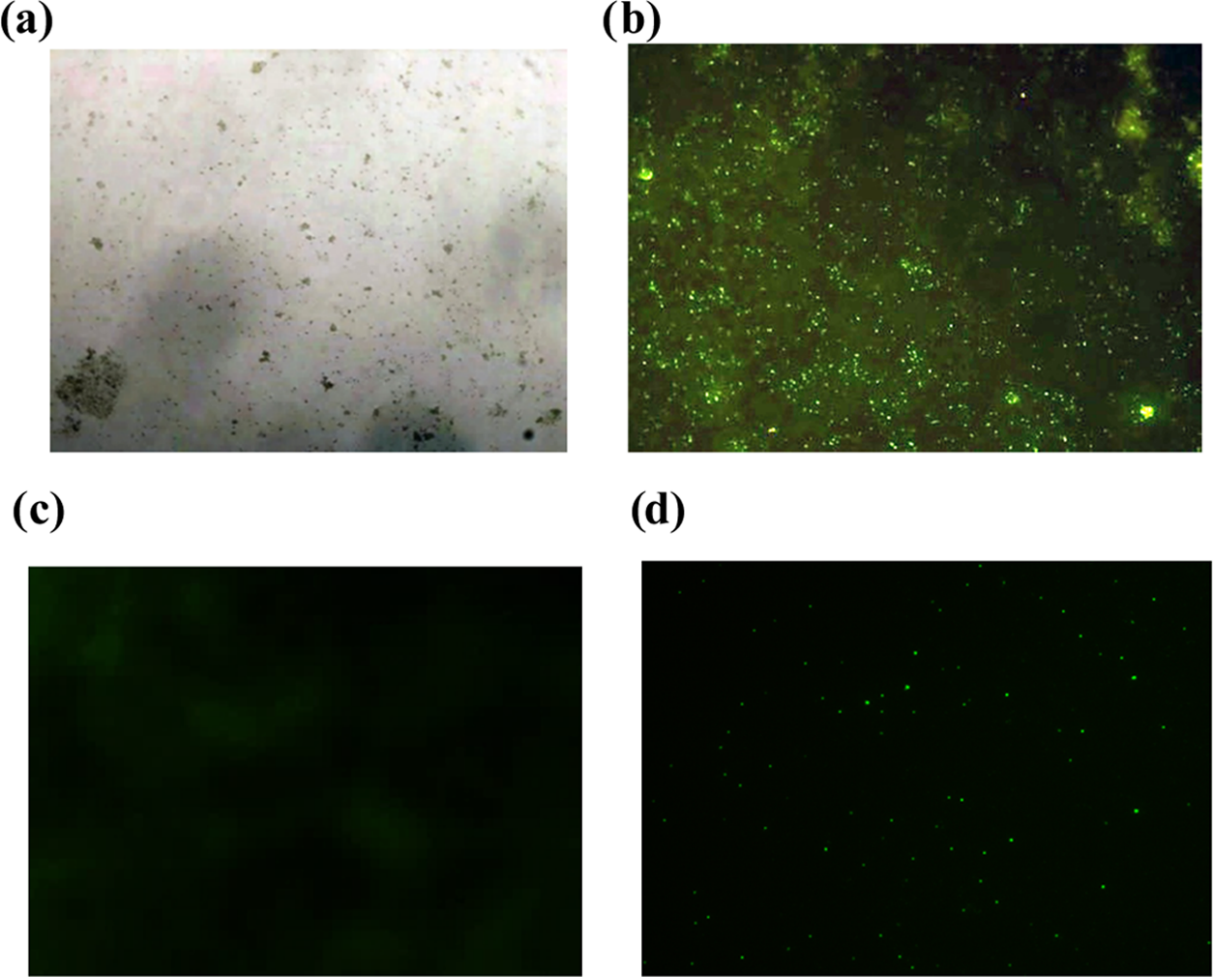
**MAnT** activity against *S. aureus* (1 × 10^8^ CFU/mL) stained with DCFH_2_-DA (5 μM). Observation in an inverted microscope (40×): (a) Clear field; (b) **MAnT** (200 μg/mL); (c) negative control (*S. aureus* with culture medium); (d) positive control (H_2_O_2_).

Figure 3 shows images of *S. aureus* cells exposed to **MAnT** and stained with the DCFH_2_-DA dye, while Figure S4 shows the other bacterial strains exposed to the compounds. Once the 2,7-DCFH_2_-DA is inside the cell, esterase enzymes release their lipophilic groups in response to oxidative metabolism, producing DCFH_2_. Upon oxidation by intracellular ROS or peroxidases, this compound is converted to the highly fluorescent green DCF. Therefore, the green coloration (as seen for example in Figure 3b,d) reveals a general state of oxidative stress in the cell, associated with the development of ROS. Together, these results indicate that **ManT** can induce a general state of oxidative stress in *S. aureus* and *E. coli* (Figures 3 and S4), while **MAcT** induces an oxidative response in *S. aureus*. From the results obtained for **MAnT** against *S. aureus*, we can deduce that oxidative stress is associated with cell death. Bacterial death is known to correlate with the appearance of ROS.^48^

As mentioned, experimental data obtained from antibacterial studies showed that tetrandrine derivatives exhibit the highest activity against *S. aureus*. The possible mechanism of action against *S. aureus* could be related to the high percentage of negatively charged phospholipids in this type of bacteria, which could interact with the cationic derivatives, damaging the membrane and making it permeable.^49,50^ In addition, Lee et al. reported that tetrandrine interacts with the peptidoglycan wall, presenting an interference in cell wall biosynthesis.^15^ Despite the promising results obtained with the tetrandrine derivatives studied in this work, further studies are still needed to clarify the mechanism of bacterial death.

### Electrophoretic Mobility Assays

**Figure 4:**
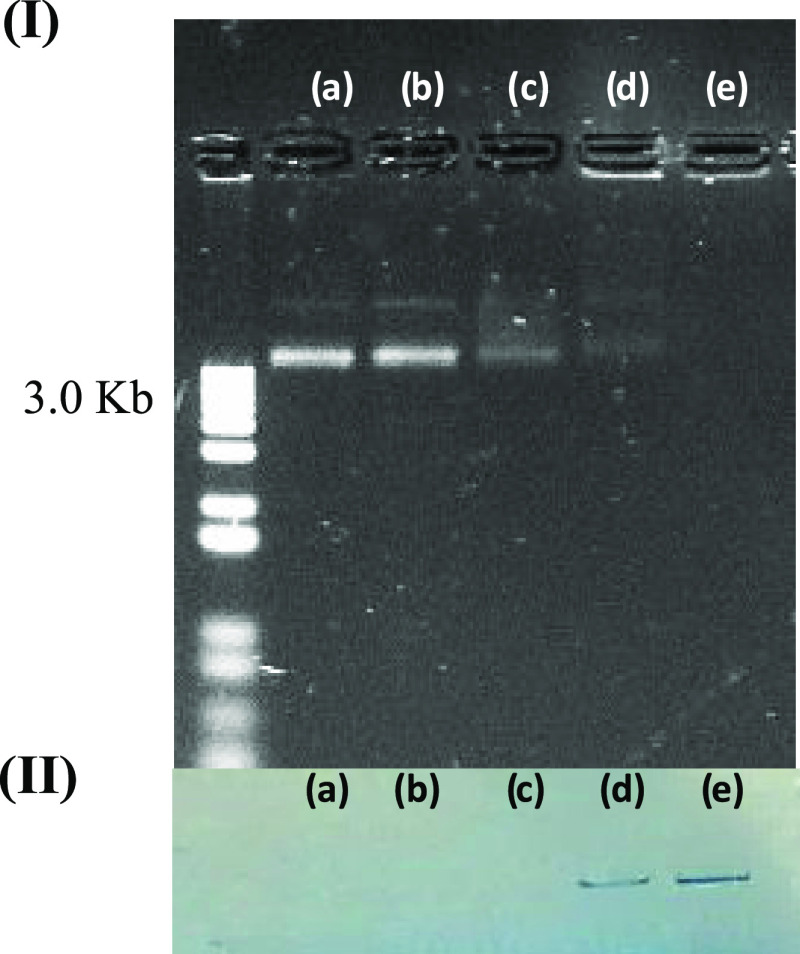
(I) Electrophoresis mobility shift assays for (a) 100 ng of bacterial plasmid DNA, and (b) samples of bacterial plasmid DNA incubated for 2 h with DMSO, or with 0.1 nmol concentration of (c) **TET**, (d) **MAcT**, and (e) **MAnT**. (II) Observation with the naked eye of the electrophoresis mobility shift assay.

The migration of DNA in agarose gels is affected by a molecular-weight-dependent mechanism in combination with the effect of the gel matrix and noncovalent interactions. Electrophoretic mobility assays can be used to qualitatively demonstrate the binding between DNA and small molecules.^51^Figure 4 illustrates clearly that natural tetrandrine and **MAcT** reduce the mobility of the bacterial plasmid DNA, while **MAnT** totally inhibits its mobility. Demonstrating that this compound has a greater interaction with the DNA.

The fact that these derivatives can bind to bacterial DNA, as well as other ds-DNA models,^32^ could imply that once these compounds enter the bacterial cell, they could interact with the DNA and cause damage. As mentioned, further studies are still needed to clarify the mechanism of bacterial death; however, these compounds have a great potential as antibacterial agents, with an outstanding activity of the derivatives against *S. aureus*.

### Free Radical Scavenging Capacity

The scavenging capacity of tetrandrine and its derivatives **MAnT** and **MAcT** was determined with the ABTS and DPPH assays (Table 2). According to the values summarized in Table 2, tetrandrine shows a higher percentage of inhibition (*p* < 0.05) on the ABTS radical (96.71 ± 0.54%) and the lowest IC_50_ (2.13 ± 0.08 μg/mL), these results are followed by those obtained with **MAnT** and **MAcT**. Similar results have been found with the related alkaloids efarantine and fangchinoline, with structural similarity to tetrandrine,^52^ which presented IC_50_ values of 7.26 and 3.90 μg/mL, respectively, these values being higher than the corresponding one obtained for the tetrandrine derivative **MAnT**. On the other hand, Makhaeva et al. (2017) reported compounds derived from acridine with IC_50_ values between 18.9 and 47.2 μM, which are significantly higher compared to the obtained for **MAnT** (5.08 ± 3.03 μM; 4.59 ± 0.20 μg/mL) but similar to that for **MAcT** (26.74 ± 0.27 μM; 23.94 ± 0.24 μg/mL) in the ABTS assay.^53^

**Table 2:** Effect of Tetrandrine (**TET**) and Its Derivatives **MAcT** and **MAnT** on the Percentage (%) of Inhibition of the Radicals ABTS^•+^ and DPPH^•^, and Its Half-Maximal Inhibitory Concentration IC_50_ (μg/mL)^a^

	% of inhibition ± SD				IC_50_ (μg/mL) ± SD			
	**TET**	**MAnT**	**MAcT**	gallic acid	**TET**	**MAnT**	**MAcT**	gallic acid
ABTS^•+^	96.71 ± 0.54^a^	92.92 ± 0.23^b^	60.85 ± 2.51^c^	99.87 ± 1.32	2.13 ± 0.08^a^	4.59 ± 0.20^b^	23.94 ± 0.24^c^	2.53 ± 0.78
DPPH^•^	3.74 ± 1.50^b^	9.32 ± 2.62^a^	8.31 ± 0.67^a^	93.34 ± 1.56	ND*	ND*	ND*	9.87 ± 1.03

a Significant differences (*p* < 0.05) are indicated by different letters (a–c), one-way ANOVA. ND* = The IC_50_ value was not obtained at the maximum concentration used. Values are mean ± standard deviation (SD) of three repetitions (*n* = 3).

The IC_50_ value for DPPH^•^ could not be determined at the concentrations used in this study. However, the results of the inhibitory activity of the ABTS^•+^ and DPPH^•^ radicals showed a great difference. In general, some authors report lower anti-free-radical activity on the ABTS^•+^ radical in pure compounds.^54,55^

Tetrandrine and its derivatives **MAnT** and **MAcT** are capable, at physiological conditions, in which their protonated dicationic forms predominate, of donating protons to stabilize the free radicals ABTS^•+^ and DPPH^•^. Free radicals (RL) possess an unpaired electron in the last orbital, which makes them unstable and highly reactive. Because they try to achieve electrochemical stability, they remove electrons from stable molecules, such as those found in the cell membrane, potentially causing cell destabilization, degradation, and oxidation. Antioxidants stabilize the RL by different mechanisms: single electron transfer (SET) and hydrogen atom transfer (HAT).^56^ As mentioned, tetrandrine and its derivatives can carry out a HAT mechanism since they have a proton that can be transferred to radicals.

However, the DPPH^•^ radical scavenging capacity of the compounds shows lower inhibition values (3–9%), probably caused by a steric hindrance of compounds, considering the reduction of DPPH^•^ is better with small compounds. Additionally, the polarity of the compounds plays an important role in the scavenging capacity. The ABTS^•+^ radical reacts with both hydrophilic and lipophilic antioxidants, while the DPPH^•^ is more selective.^57^ Tetrandrine could be considered more of a lipophilic compound, while **MAnT** and **MAcT** are amphiphilic. However, depending on the pH conditions, all of them could have both nitrogen atoms positively charged. This could explain the higher affinity of these compounds for the ABTS^•+^ radical.

In general, the antioxidant properties of compounds have been related to their ability to prevent or treat diseases. In a previous work, we showed that **MAnT** has a high cytotoxic activity against tumoral cell lines.^32^ Taking into account that **MAnT** also showed the highest antibacterial activity, it could be considered as a compound with a high biological activity.

### Cyclic Voltammetry

Cyclic voltammetry studies were performed to characterize the redox-active **MAnT** and **MAcT** tetrandrine derivatives (Figures 1 and S5). Glassy carbon electrodes containing the different derivatives adsorbed at the surface were analyzed in deoxygenated DMSO solutions, with 0.20 M NBu_4_PF_6_ as electrolyte, over a potential range of 0.00 to −2.20 V versus Ag/AgCl, using a scan rate of 0.100 V/s.

The electrochemical reduction of adsorbed **MAnT** at the glassy carbon electrode surface shows one cathodic peak (I) at −2.033 V and the corresponding oxidation peak at −1.960 V. These peaks are reversible, which is evidenced by the value of the peak current ratio (*I*_pa_/*I*_pc_) close to unity and involve the reduction/oxidation of the substituent anthracene on the tetrandrine derivative; see Table 3. On the other hand, the tetrandrine derivative **MAcT**, which carries a heteroaromatic substituent, shows a cathodic peak (I) at −0.895 V, indicating that its reduction process requires less energy (Table 3). In other words, **MAcT** has the greatest reduction potential, and this capacity is reversed in the direction of oxidation; consequently, **MAnT** requires less energy for its oxidation, which may explain its better antioxidant activity. Furthermore, in the **MAcT** voltammogram, there is no oxidation peak. Although these results are in agreement with the antioxidant activity of tetrandrine derivatives, they do not correlate with their antibacterial activity.

**Table 3:** Electrochemical Reduction (*E*_pc_), Oxidation (*E*_pa_), and Half-Wave (*E*_1/2_) Potentials as well as Δ*E*_p_ (*E*_pc_–*E*_pa_) and Current Ratio (*I*_pa_/*I*_pc_) Values for the Tetrandrine Derivatives **MAnT** and **MAcT**, Determined from Cyclic Voltammetry Experiments at 25 °C in DMSO with 0.20 M NBu_4_PF_6_, Scan Rate 0.1 V/s

compound	peak	*E*_pc_/V	*E*_pa_/V	Δ*E*_p_/V	*E*_1/2_/V	*I*_pc_/μA	*I*_pa_/μA	*I*_pa_/*I*_pc_
**MAnT**	I	–2.033	–1.960	0.073	–1.997	–5.53	4.52	0.817
**MAcT**	II	–0.895				–5.86		

Regarding the latter, for example, it has been reported for a library of naphthoquinones that redox behavior may be a significant factor in determining antibacterial activity.^48^ On the other hand, it is well known that the antibacterial mechanism of natural alkaloids may be related to the disruption of the bacterial cell membrane (inhibition of bacterial cell wall synthesis and change in cell membrane permeability), damage to the DNA function, and inhibition of bacterial metabolism and protein synthesis.^6^ For these reasons, the biological activity of the tetrandrine derivatives may be attributed to different factors, including their better DNA-binding ability in comparison to natural tetrandrine. In this sense, transcriptomic analysis could be a valuable tool to reveal the specific biological processes affected in bacteria by the presence of tetrandrine derivatives. In particular, the regulation of target genes related to the membrane, DNA damage, and other stress responses caused by the compounds could be studied.^58^ Finally, the electrochemical behavior of tetrandrine was also studied, but the results were not clear.

## Conclusions

In this work, the antibacterial and antioxidant properties of tetrandrine and its mono-*N*-substituted derivatives **MAnT** and **MAcT** were explored. The results have shown that the derivatives have antibacterial properties, with very good activity against Gram-positive bacterium *S. aureus*, highlighted in the case of **MAnT**. In addition, it was shown that these derivatives damage the bacterial membrane and produce reactive oxygen species in *S. aureus* that could be responsible for the loss of viability due to the oxidative stress generated. The antioxidant capacity was also studied, showing that **MAnT** has good activity against the ABTS^•+^ radical. Furthermore, these derivatives bind to bacterial DNA, as evidenced by agarose gel electrophoresis, which could also be related to its antibacterial activity.

The results found in this work reveal that chemical modifications in natural tetrandrine improve its biological activity. We are currently working on obtaining new tetrandrine derivatives and developing deep knowledge of the biological and physicochemical properties of these compounds with good potential for biomedical and pharmaceutical applications.

## Experimental Section

### Reagents and Tetrandrine Derivative Compounds

*S*,*S*-(+)-Tetrandrine and all reagents were purchased from Sigma-Aldrich and used without further purification. Mono-*N*-substituted Tetrandrine derivatives **MAnT** and **MAcT** were prepared following the synthetic procedures reported previously.^32,33^

### Preparation and Standardization of Inoculum

McFarland standard curve was prepared from a solution containing 99.5 mL of 1% (v/v) sulfuric acid and 0.5 mL of 1.175% (w/v) barium chloride in distilled water. The optical density at 600 nm (OD_600_) was measured for different dilutions and used for comparison of bacterial suspension with standard. To prepare inoculums, colonies from a pure culture of each tested bacterium were transferred to Brain-Heart Infusion (BHI, DIFCO) broth and incubated at 35–37 °C for 18–24 h. The turbidity of the culture was compared with the 0.5 McFarland Nephelometer standard to get 1 × 10^8^ CFU/mL.^59^

### Antibacterial Activity

The natural tetrandrine and its derivatives were tested as antibacterial agents against *E. coli* (ATCC 25922), *S. aureus* (ATCC 25923), *K. pneumoniae* (ATCC 13883), and *P. aeruginosa* (ATCC 10145) of the American Type Culture Collection (ATCC). Bacterial susceptibility testing was performed using an adaptation of the standard broth microdilution assay recommended by the Institute of Clinical and Laboratory Standards (CLSI).^15^ BHI culture media with DMSO, ampicillin trihydrate (A6140, Sigma-Aldrich), and chloramphenicol (C0378, Sigma-Aldrich) were used as negative and positive control samples.

Solutions of the compounds were prepared in DMSO and subsequently diluted 1:10 with a BHI culture medium. A volume of 150 μL of this solution containing different concentrations of tetrandrine or its derivatives was added to wells in a 96-well microtiter plate and mixed with 50 μL of bacterial suspension (1 × 10^8^ CFU/mL). The final concentrations of the compounds ranged from 200 to 6.25 μg/mL. Plates were incubated at 37 °C for 24 h. The OD_600_ values for each well were determined with a Multiskan microplate reader (Thermo Scientific, Inc. Multiskan GO, NY). Then, 15 μL per well of the resazurin dye (0.02%) was added and the microplate was incubated for 4 h at 37 °C, and the OD_600_ values were determined again.^43,44^

The experimental data were estimated by Probit survival/reliability test (NCSS ver. Ten statistical software, NCSS LLC, Kaysville, UT) to obtain the concentrations of tetrandrine and its derivatives that inhibited 50% (IC_50_) and 99% (IC_99_), and the MIC (minimum inhibitory concentration) was established as the minimum concentration where inhibition began (first percentile).

### Analysis of Cell Membrane Integrity and Oxidative Stress by Fluorescence Microscopy

Observations were made in an inverted microscope (Model DMi8, Leica Microsystems, Germany) equipped with fluorescence filters (546/10 RHOD excitation filter and emission 585/40, 350/50 DAPI filter and emission 460/40, and 480/40 FITC filter and 527/30 emission), a cooled monochromatic DFC 450C camera (Leica Microsystems, Germany), and fluorescence overlay software (LAS AF ver. 3.1.0, Leica Microsystems CMS GmbH, Germany).

Solutions of the compounds were prepared in DMSO and then diluted 1:10 with BHI culture medium obtaining a concentration of 200 μg/mL. This solution (150 μL) was added to wells in a 96-well microtiter plate and 50 μL of bacterial suspension (1 × 10^8^ CFU/mL). The plates were incubated at 37 °C for 24 h, and the supernatant was removed and stained with the fluorescent dyes.

### Cell Membrane Integrity

Propidium iodide (PI) fluorescent staining for nucleic acids was employed to visualize the integrity of the bacterial membrane. Two drops of 3 μM PI solution were added to each well, and the plates were incubated for 15 min under refrigeration in the dark. This dye is excluded from the cell membrane when it maintains its integrity. However, when the cell suffers a membrane damage or when the integrity of its membrane is compromised due to various factors, propidium iodide intercalates into double-stranded DNA (ds-DNA) and thus shows a red color.^60^

### Detection of Reactive Oxygen Species (ROS) in Bacteria

2′,7′-Dichlorodihydrofluorescein diacetate (2,7-DCFH_2_-DA) is a nonfluorescent cell-permeable dye that is hydrolyzed intracellularly to its polar but nonfluorescent form DCFH_2_, on the action of cellular esterase enzymes and thus is retained in the cell. Oxidation of DCFH_2_ by the action of intracellular reactive oxygen species (ROS) and other peroxides turns the molecule into its highly fluorescent form (DCF) that can be detected by various fluorescent methods.^60^ Considering this mentioned, the 2,7-DCFH_2_-DA dye was employed according to the methodology of Rajneesh Pathak et al., with some modifications.^5,61^ Two drops of a 5 μM solution of 2,7-DCFH_2_-DA were added to each well, and the plates were incubated for 1 h under refrigeration in the dark. *S. aureus* (1 × 10^8^ CFU/mL) in culture medium with DMSO was used as a negative control, and *S. aureus* with 0.001 M H_2_O_2_ was used as a positive control.

### Free Radical Scavenging Capacity

#### 2,2′-Azino-bis-(3-ethylbenzothiazoline-6-sulfonic Acid) (ABTS^•+^) Method

The natural tetrandrine and their derivatives were studied against the radical ABTS^•+^ according to the modified methodology of Re et al.^62^ The ABTS^•+^ monocation radical was prepared by dissolving 19.3 mg of ABTS in water (5 mL) and then adding an amount of 88 μL of a solution of K_2_S_2_O_8_ (37.8 mg/mL) to generate the working reagent. The mixture was incubated in the dark for 16 h at room temperature. Later, 1 mL of this solution was dissolved in 88 mL of ethanol. The absorbance was adjusted to 0.7 using a microplate reader of 96 wells (Thermo Fisher Scientific, Inc. Multiskan GO, NY) at 734 nm. The compounds (20 μL) with concentrations from 1 to 30 μg/mL were mixed with an ABTS^•+^ radical solution (270 μL). Samples were incubated for 30 min at room temperature and then measured in a microplate reader. The results were reported as % of inhibition of the radical according to eq 1. Moreover, the half-maximum inhibitory concentration (IC_50_) was determined. Methanol was used as a negative control, and gallic acid was used as a positive control.
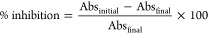
1where Abs_initial_ is the absorbance of the blank and Abs_final_ is the absorbance of the compound with the radical.

#### 1,1′-Diphenyl-2-picrylhydrazyl (DPPH•) Method

DPPH radical (1.25 mg/mL) was prepared in methanol, and the solution was adjusted to 0.7 at 515 nm.^63^ Tetrandrine and its derivatives (20 μL) were mixed with the DPPH^•^ radical solution (200 μL). The concentrations of each compound were evaluated from 1 to 30 μg/mL. All of the solutions were incubated for 30 min at room temperature and then measured in a microplate reader. This experiment was carried out in triplicate, and the results were reported in % of inhibition of the radical (eq 1). Methanol was used as a negative control, and gallic acid was used as a positive control.

### Electrophoretic Mobility Shift Assays

100 ng samples of plasmid DNA pCR 2.1 (Thermo Scientific) containing a cloned DNA sequence of a total of 4.5 kb were combined with 100 μM tetrandrine or its derivatives, **MAnT** or **MAcT**, in a solvent mixture of DMSO and deionized water 10:90 (v/v) and incubated for 2 h at 37 °C. The samples were loaded into 1% agarose gel, and its electrophoretic mobility was examined for 30 min at 60 V. After staining the gel under gentle agitation for 5 min with ethidium bromide (5 μg/mL), the samples were washed with water for 2 h and then revealed with UV light using a Gel Doc EZ documentation system from Bio-Rad.^64^ The solvent mixture and a solution of the plasmid were used as controls.

### Cyclic Voltammetry

Cyclic voltammograms (CV) were obtained with a conventional three-electrode cell, with the auxiliary electrode being a platinum wire and the reference electrode Ag/AgCl (KBr, 3 M). Experiments were carried out in dimethyl sulfoxide (DMSO) solutions containing 0.20 M tetrabutylammonium hexafluorophosphate (NBu_4_PF_6_) as supporting electrolyte, using a Metrohm PGSTAT 128N equipment. Prior to the measurements, the working electrodes were prepared by evaporating a drop (5.0 μL) of a suspension of the corresponding tetrandrine derivative in acetonitrile (1 mg/mL) placed on a glassy carbon electrode (GCE, BAS MF 4012, exposure area 0.071 cm^2^). CVs were collected at room temperature with scan rates of 100 mV/s with *iR* compensation.^65^

## Data Availability

The data that support the findings of this study are available in the Supporting Information.

## Supporting Information Available

Graphs of bacterial growth in CFU/mL × 10^8^ against the concentration of tetrandrine and its derivatives, **MAnT** and **MAcT**; results of their antibacterial activity in IC_50_/IC_99_ values and photographs of the microplate cultures of the different bacterial strains (1 × 10^8^ CFU/mL) with resazurin; photographs of the integrity of the cell membrane (propidium iodide staining) and detection of reactive oxygen species in bacteria (2,7-DCFH2-DA staining) studies of the bacterial strains incubated with tetrandrine and its derivatives; and cyclic voltammograms of **MAnT** and **MAcT** (PDF)
