# Supplementary material for: Tetrandrine Derivatives as Promising Antibacterial Agents
Source: ACS Omega. 2023 Jul 26;8(31):28156–64. doi: 10.1021/acsomega.3c01368 (PMC10413380; doi:10.1021/acsomega.3c01368)
Supplement: Supplementary file 1 — ao3c01368_si_001.pdf [file ao3c01368_si_001.pdf]

Supporting Information

"Tetrandrine Derivatives as Promising Antibacterial Agents"

Viviana I. Calvillo-Páez,[1] Maribel Plascencia-Jatomea,[2] Adrián Ochoa-Terán,[1*] Carmen L. Del-Toro-Sánchez,[2] Ricardo I. González-Vega,[2] Sandra M. González-Martínez,[3] and Karen Ochoa Lara[3]*

[1] Centro de Graduados e Investigación en Química, Tecnológico Nacional de México, Campus Tijuana, CP 22444. Tijuana, B. C., México. *Corresponding author. E-mail: adrian.ochoa@tectijuana.edu.mx

[2] Departamento de Investigación y Posgrado en Alimentos, Universidad de Sonora, Rosales y Encinas s/n, Col. Centro CP 83000. Hermosillo, Sonora, México.

[3] Departamento de Investigación en Polímeros y Materiales, Universidad de Sonora, Rosales y Encinas s/n, Col. Centro CP 83000. Hermosillo, Sonora, México. *Corresponding author. E-mail: karen.ochoa@unison.mx, karenol@polimeros.uson.mx

Table S1. Required concentration (μg/mL and μM) of tetrandrine and its derivatives to inhibit the growth of ATTC bacterial strains by 50% and 99% (IC_{50} and IC_{99}).

	IC_{50} μg/mL ± SD (IC_{99} μg/mL ± SD)<br>**IC_{50} μM ± SD (IC_{99} μM ± SD)**			
	S. aureus	*E. coli*	*K. pneumoniae*	*P. aeruginosa*
**MAcT**	22.01 ± 0.71[a]<br>(79.36 ± 6.05)<br>**24.60 ± 0.80**<br>**(88.69 ± 6.84)**	ND*	78.39 ± 4.97[b]<br>(>200)<br>**87.61 ± 5.62**	ND**
**MAnT**	1.51 ± 0.45[b]<br>(64.19 ± 14.77)<br>**1.68 ± 0.51**<br>**(71.68 ± 16.98)**	61.51 ± 1.41[a]<br>(160.95 ± 8.62)<br>**68.69 ± 1.62**<br>**(179.74 ± 9.91)**	30.19 ± 1.06[a]<br>(134.37 ±11.07)<br>**33.71 ± 1.21**<br>**(150.06 ± 12.7)**	ND**
**Tetrandrine**	ND*	ND*	ND*	ND**

Significant differences ($p < 0.05$) are indicated by different letters (a-b), one-way ANOVA.
Values are mean ± standard deviation (SD) of three repetitions (*n=3*).
ND*= The IC_{50} value was not obtained at the maximum concentration used (200 μg/ml).
ND** The variation of the data obtained did not allow the calculation of the IC_{50}, but the percentages of inhibition obtained experimentally indicate that the IC_{50} are above the maximum tested (200 μg/ml).

a) *S. aureus*

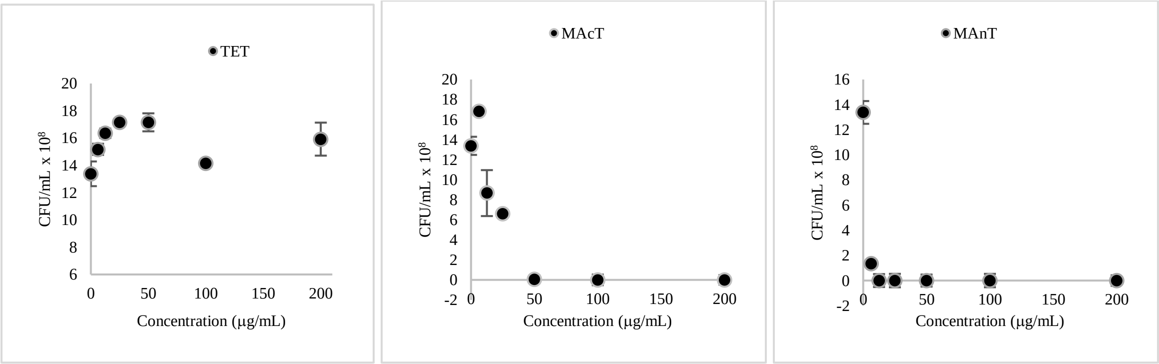

b) *E. coli*

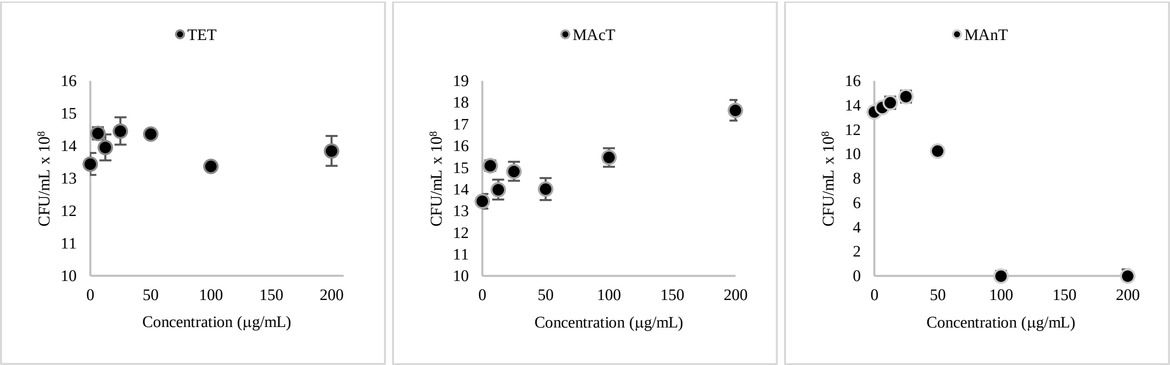

c) *K. pneumoniae*

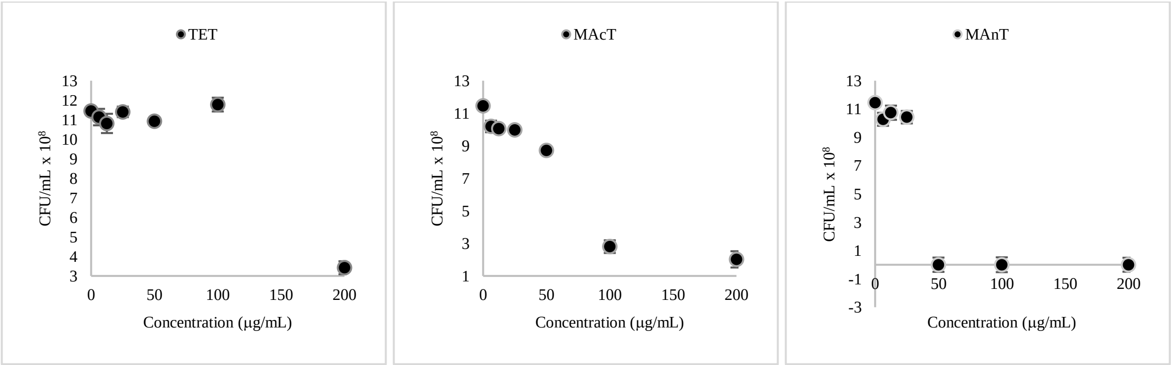

d) *P. aeruginosa*

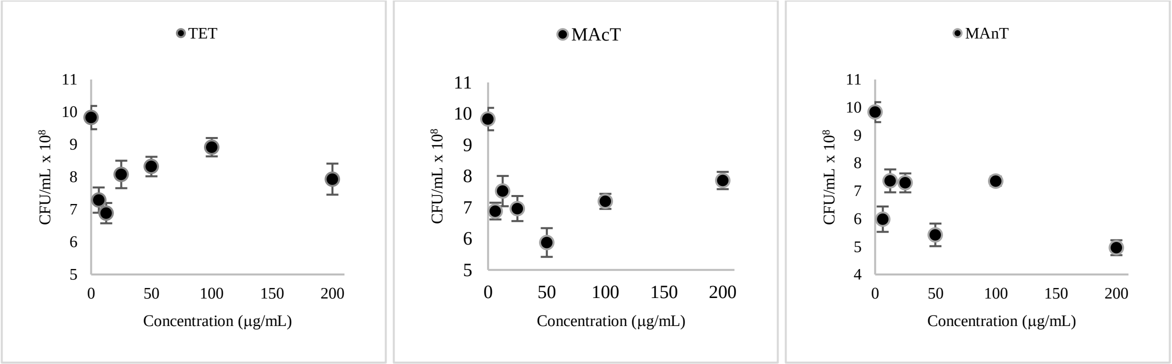

Figure S1. Graphic of the concentration (µg/mL) of tetrandrine and its derivatives, **MAnT** and **MAcT**, *vs* bacterial growth in CFU/mLx10^8 for strains of: **a)** *S. aureus*; **b)** *E. coli;* **c)** *K. pneumoniae* and **d)** *P. aeruginosa*.

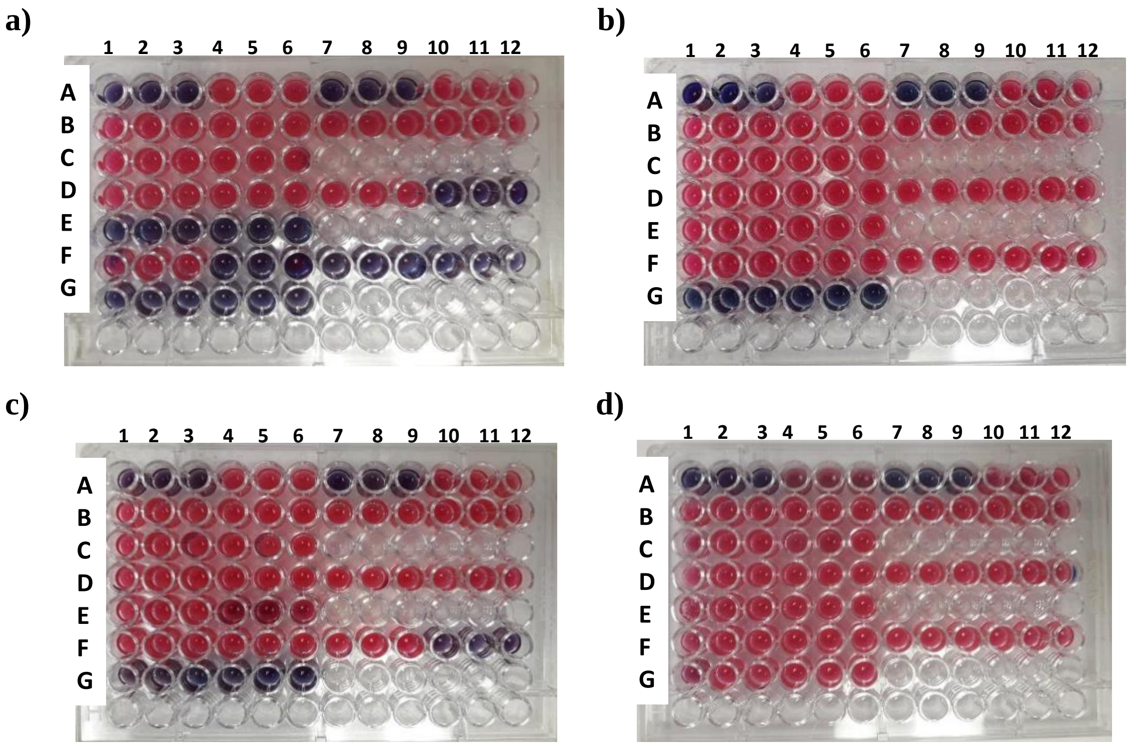

Figure S2. Microplate cultures of the different bacterial strains (1x10^8 CFU/mL), with resazurin: a) *S. aureus*, b) *E. coli*, c) *K. pneumoniae*, and d) *P. aeruginosa*. In all cases: **A1-A3** wells: BHI medium, **A4-A6**: BHI + respective inoculum, **A7-A9**: BHI + DMSO, **A10-A12:** BHI + respective inoculum + DMSO, **B1-C6:** Tetrandrine at 6.25, 12.5, 25, 50, 100 and 200 µg / mL, **D1-E6: MAcT** at 6.25, 12.5, 25, 50, 100 and 200 µg/mL, **F1-G6**: **MAnT** at 6.25, 12.5, 25, 50, 100 and 200 µg/mL.

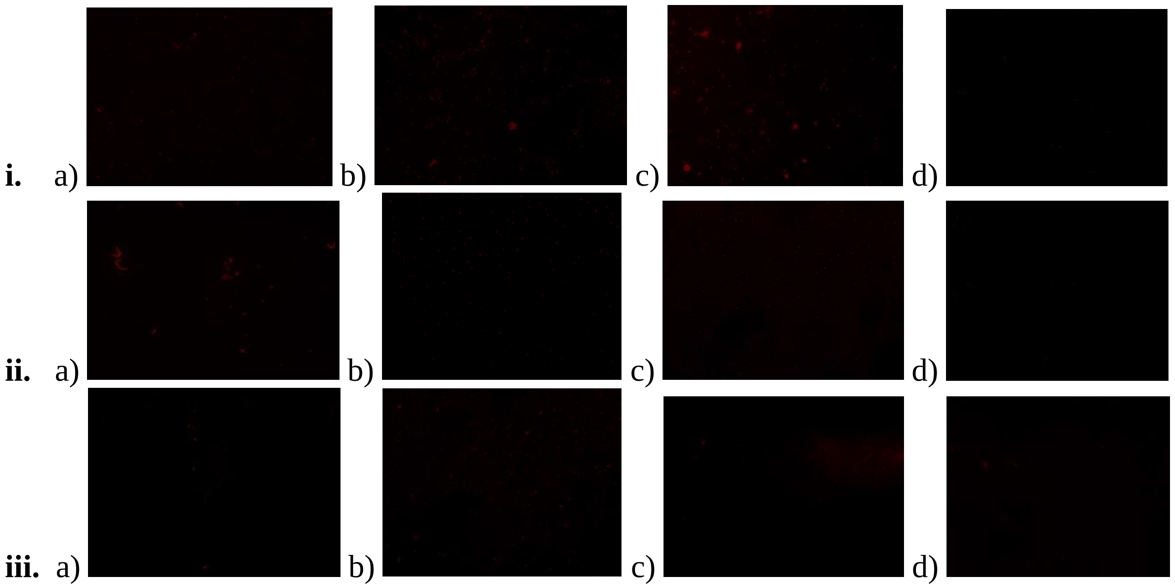

Figure S3. Photographs taken with a 40x inverted microscope of the bacterial strains ($1x10^8$ CFU/mL) **i)** *E. coli*, **ii)** *K. pneumoniae*, **iii)** *P. aeruginosa* incubated 24 hours in BHI medium with a) tetrandrine (200 µg/mL), b) **MAcT** (200 µg/mL), c) **MAnT** (200 µg/mL), d) control (no added compound); all stained with propidium iodide.

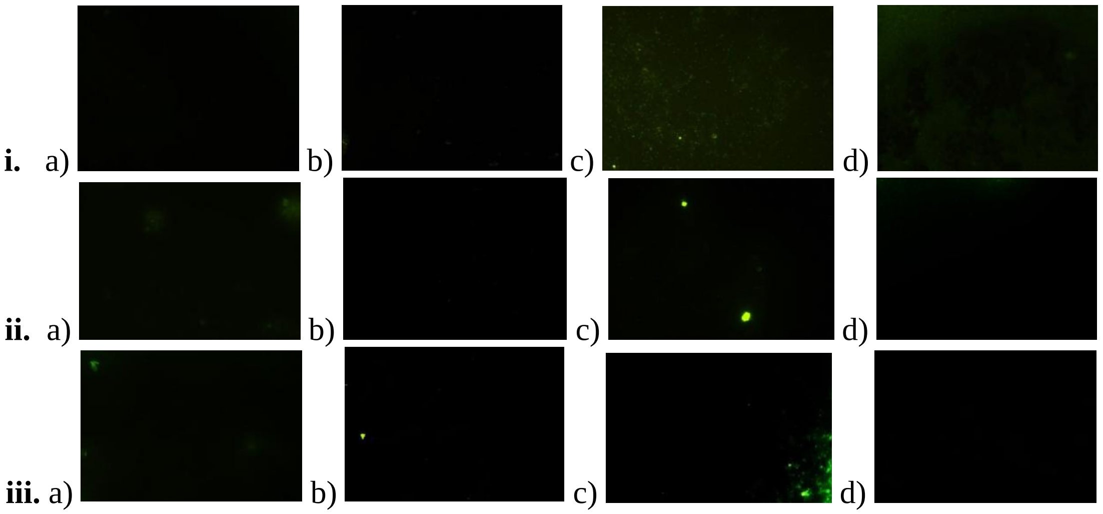

Figure S4. Photographs taken with a 40x inverted microscope of the bacterial strains ($1x10^8$ CFU/mL) **i)** *E. coli*, **ii)** *K. pneumoniae*, **iii)** *P. aeruginosa* incubated 24 hours in BHI medium with a) tetrandrine (200 µg/mL), b) **MAcT** (200 µg/mL), c) **MAnT** (200 µg/mL), d) control (no added compound); all stained with $DCFH_2$-DA.

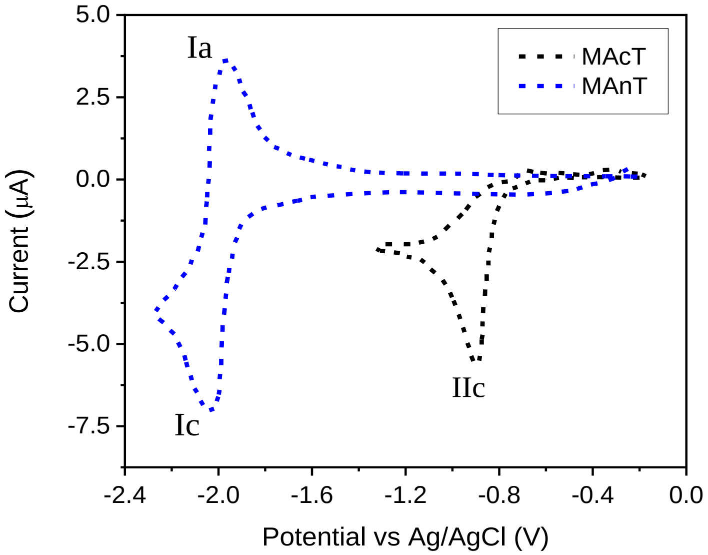

Figure S5. Cyclic voltammetry of the tetrandrine derivatives **MAnT** and **MAcT** deposited on glassy carbon electrodes (drop dry method), in DMSO with 0.20 M NBu_4PF_6, scan rate 0.100 V/s.
